# Supplementary material for: Self-organizing glycolytic waves tune cellular metabolic states and fuel cancer progression
Source: Nat Commun. 2025 Jul 1;16:5563. doi: 10.1038/s41467-025-60596-6 (PMC12217304; doi:10.1038/s41467-025-60596-6)
Supplement: Supplementary file 1 — Supplementary Information [file 41467_2025_60596_MOESM1_ESM.pdf]

Supplementary Fig. 1

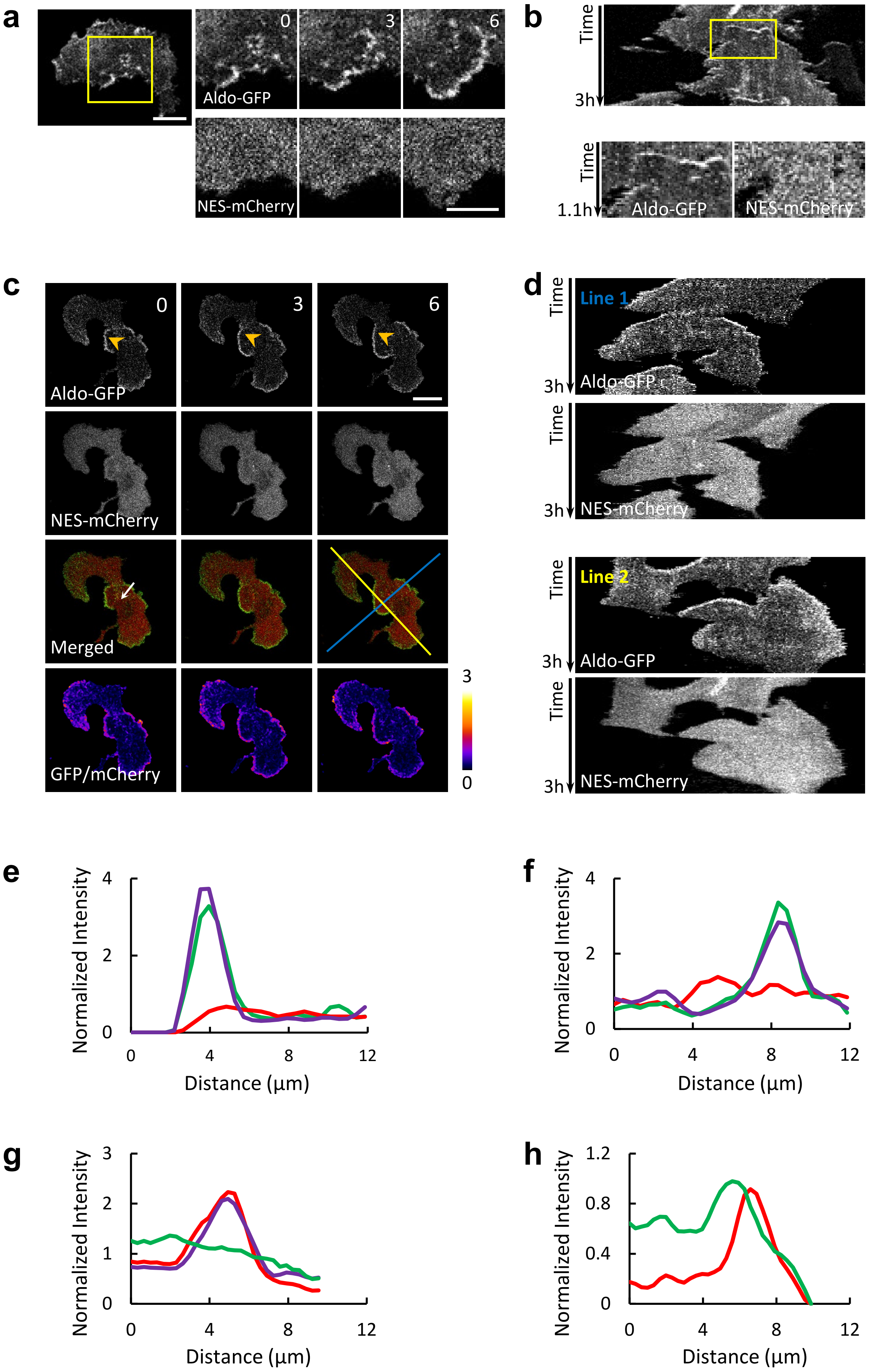

Supplementary Fig. 1 | Enrichment of aldolase in F-actin waves and protrusions.

(**a**) A zoomed-in view of the wave in the cell shown in **Fig. 1a.** Scale bar: 20 μm.

(**b**) A zoomed-in view of the kymographs shown in **Fig. 1b.**

(**c**) Another example of an MCF-10A M3 cell expressing aldolase-GFP and NES-mCherry similar to **Fig. 1a**. Scale bar: 20 μm. The orange arrowheads indicate expanding waves propagating across the basal surface of the cell.

(**d**) Kymographs along the two lines in (c) over 3 h.

(**e**) Normalized intensity of aldolase-GFP (green), NES-mCherry (red), and the GFP/mCherry ratio (purple) across the white arrow in (c).

(**f**) Normalized intensity of aldolase-GFP (green), NES-mCherry (red), and the GFP/mCherry ratio (purple) across the white arrow in **Fig. 1a**.

(**g**) Normalized intensity of NES-GFP (green), LifeAct-RFP (red), and the RFP/GFP ratio (purple) across the white arrow in **Fig. 1c**.

(**h**) Normalized intensity of aldolase-GFP (green) and LifeAct-RFP (red) across the white arrow in **Fig. 1e**.

Supplementary Fig. 2

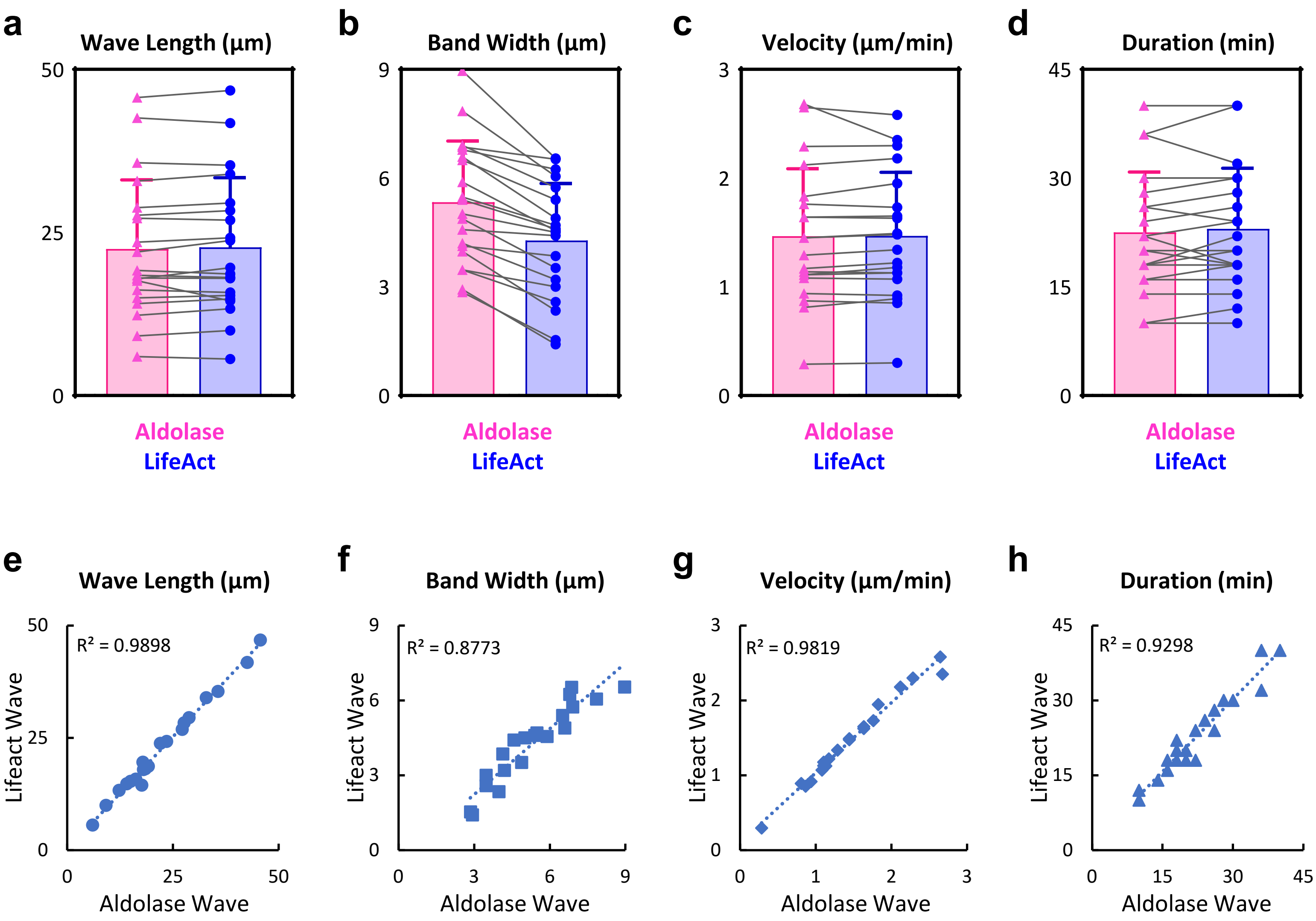

Supplementary Fig. 2 | Aldolase waves are strongly correlated with LifeAct-labeled actin waves.

(**a-d**) The mean ± SD of wave length (maximum lateral expansion) (a), band width (b), velocity (c), and duration (d) is shown for coordinated Aldolase and LifeAct waves (indicated with connecting lines) in MCF-10A-M3 cells expressing Aldolase-GFP and LifeAct-RFP. A total of 20 wave events were quantified from 8 different cells from 3 independent experiments.

(**e-h**) Correlation of wave length (e), band width (f), velocity (g), and duration (h) is shown for coordinated Aldolase-GFP and LifeAct-RFP waves in (a-d).

Supplementary Fig. 3

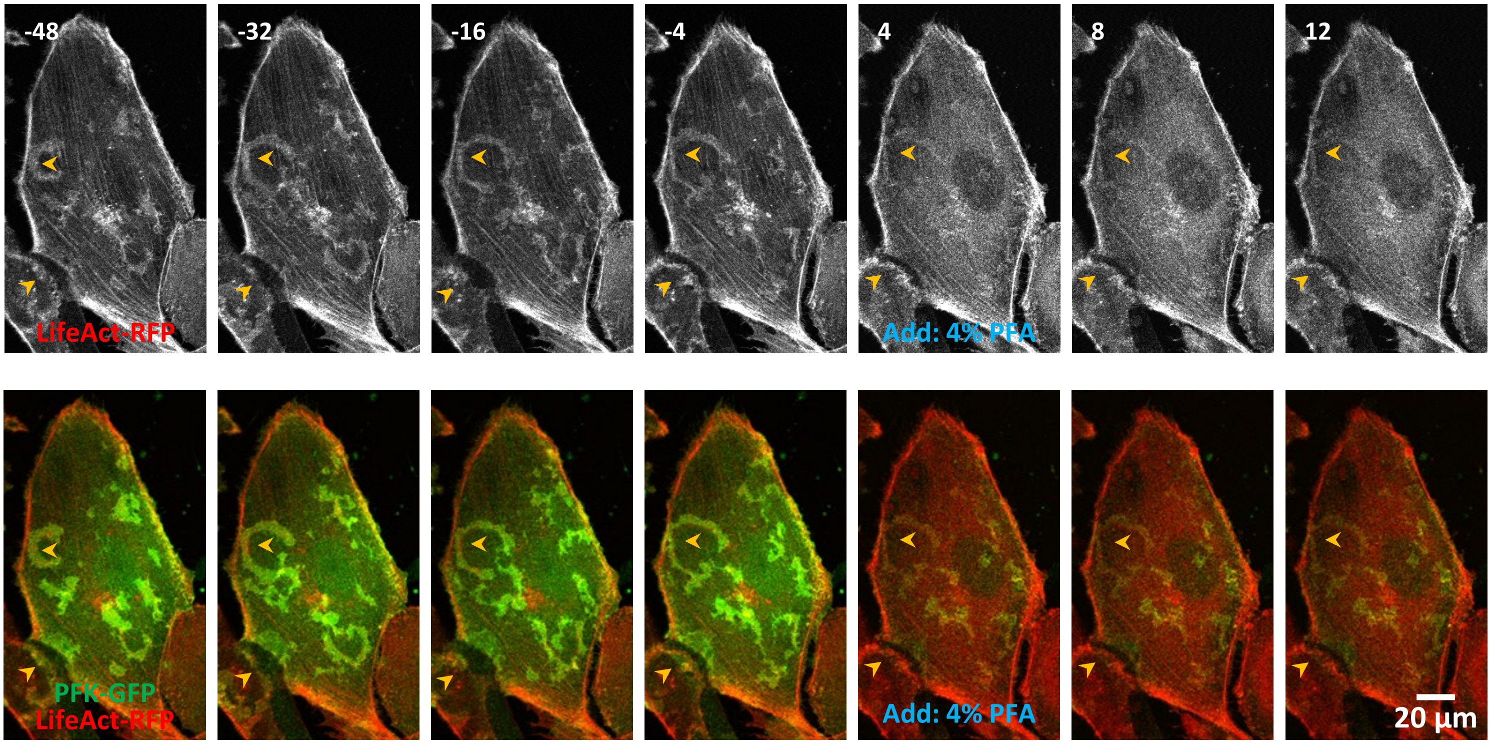

Supplementary Fig. 3 | Glycolytic and actin waves are preserved by cell fixation.

Time-lapse confocal images showing the basal surface of MCF-10A-M3 cells expressing LifeAct-RFP and PFK-GFP (shown in **Fig. 3a**) before and after fixation by 4% PFA (added at time 0). Merged images of PFK-GFP (green) and LifeAct-RFP (red) are also shown. Yellow arrow heads indicate examples of waves. Time stamp is minute. Fluorescence signals for both PFK-GFP and LifeAct-RFP after fixation were multiplied by 2 for better presentation. This shown image represents a typical example of cells from N $\geq$ 3 independent experiments.

Supplementary Fig. 4

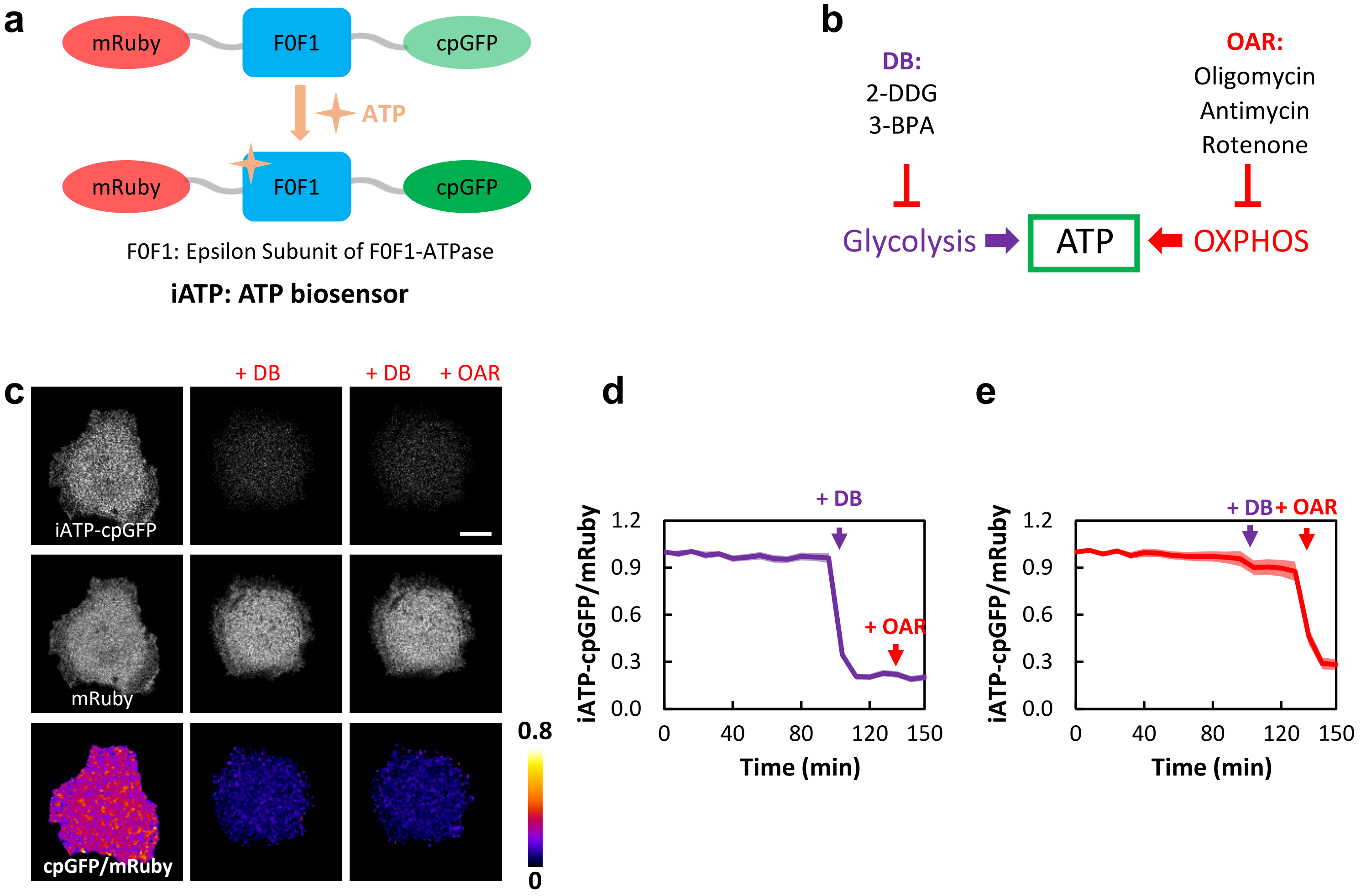

Supplementary Fig. 4 | Effects of glycolysis and OXPHOS inhibition on ATP levels.

(**a**) Design of the iATP biosensor [19]. Binding of ATP to the epsilon subunit of F0F1-ATPase causes a conformational change of the circularly permuted GFP (cpGFP), leading to increased fluorescence; while the fluorescence intensity of mRuby is not changed, serving as an internal control for the biosensor. Images were created in PowerPoint using built-in elements available within the software.

(**b**) Inhibitors for glycolysis and OXPHOS used in this study. 2-DDG: 2-Deoxy-D-glucose (10 mM), 3-BPA: 3-Bromopyruvic acid (50 μM), Oligomycin (5 μM), Antimycin A (1 μM), Rotenone (1 μM).

(**c**) Confocal images of cpGFP, mRuby, and the cpGFP/mRuby ratio of an MCF-10A M3 cell expressing the iATP biosensor treated with DB and OAR (also see **Supplementary Movie 10**). Scale bar: 20 μm.

(**d**) Plot of normalized iATP cpGFP/mRuby (mean ± SEM) over time of 27 cells from 5 independent experiments treated with DB followed by OAR at the indicated time.

(**e**) Plot of normalized iATP cpGFP/mRuby (mean ± SEM) over time of 20 cells from 5 independent experiments treated with OAR followed by DB at the indicated time.

Supplementary Fig. 5

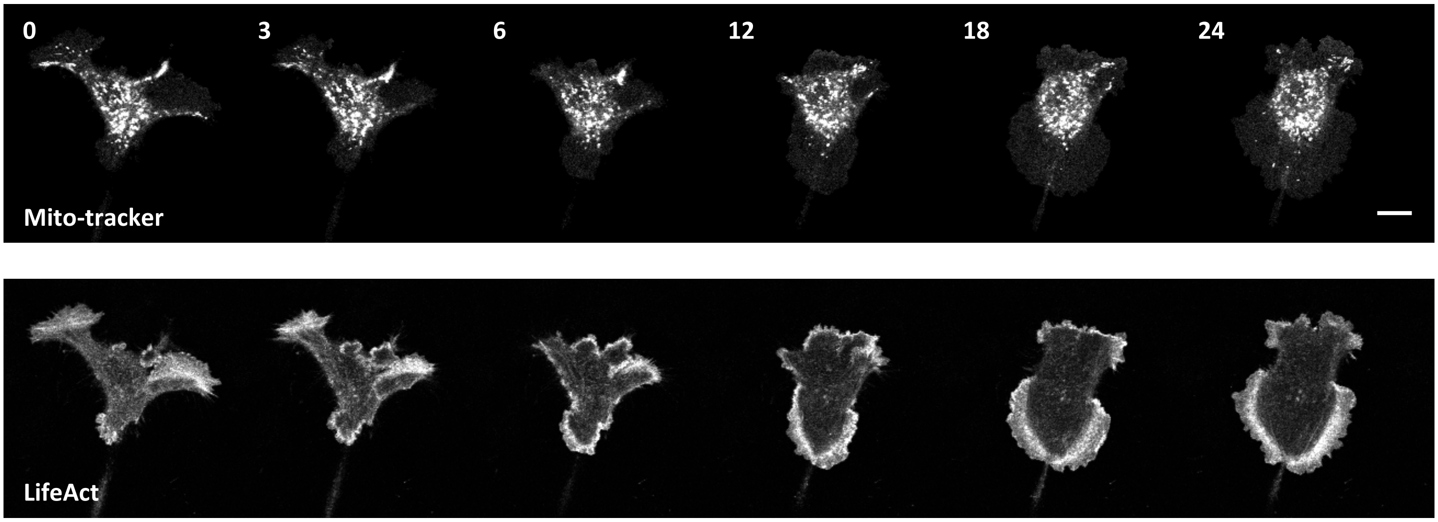

Supplementary Fig. 5 | Absence of mitochondria from the waves and protrusions.

Time-lapse confocal images of an MCF-10A M3 cell expressing Mito-tracker-Green and LifeAct-iRFP. Scale bar: 20 µm. These shown images represent a typical example of cells from N ≥ 3 independent experiments.

Supplementary Fig. 6

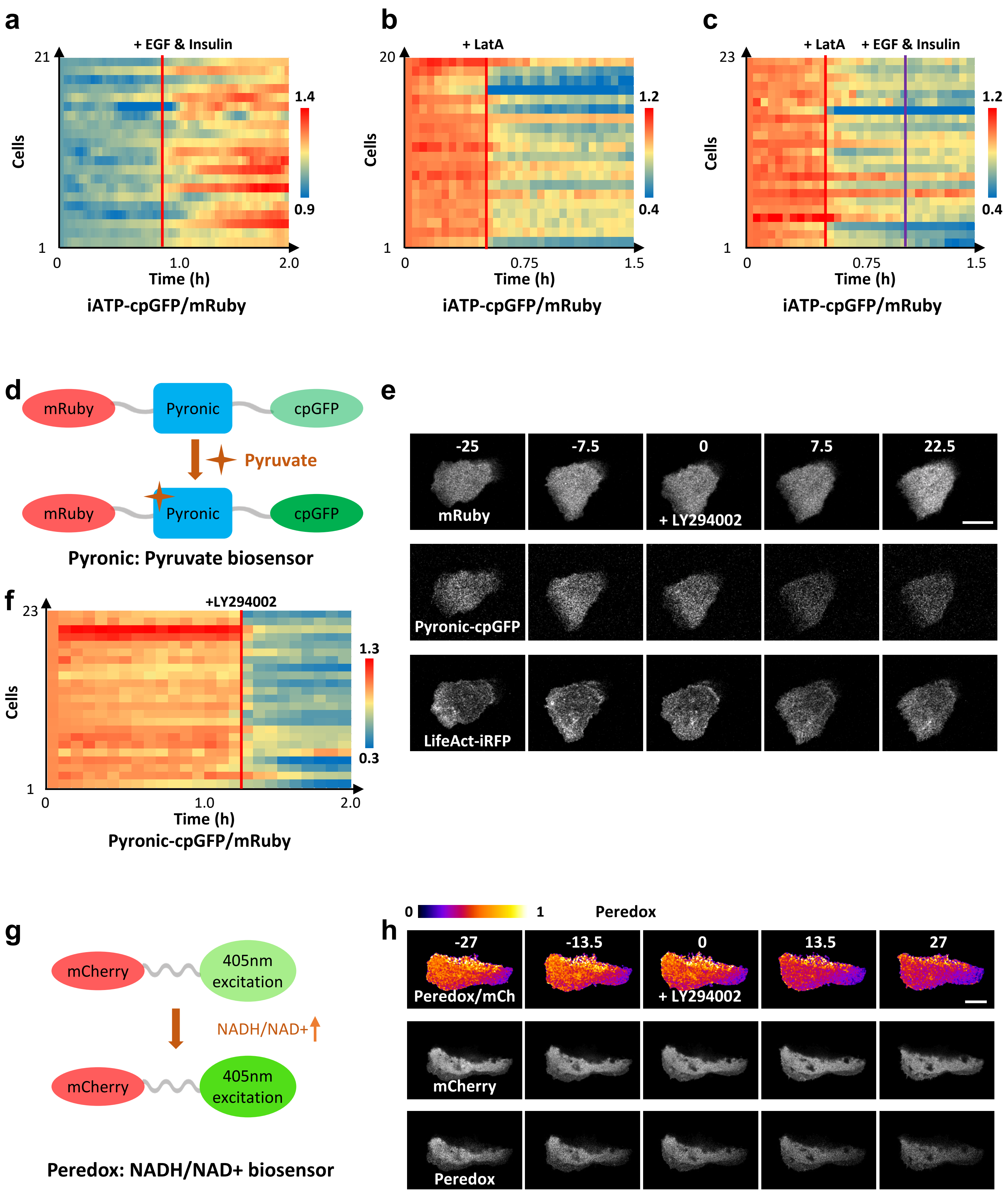

Supplementary Fig. 6 | Effects of perturbing wave activities on ATP, pyruvate, and NADH/NAD+ levels.

(**a**) Plots of iATP cpGFP/mRuby ratio in 21 cells stimulated with EGF and insulin in **Fig. 4h**.

(**b**) Plots of iATP cpGFP/mRuby ratio in 20 cells treated with Latrunculin A in **Fig. 4k**.

(**c**) Plots of iATP cpGFP/mRuby ratio in 23 cells upon treatment with Latrunculin A followed by EGF and insulin in **Fig. 4l**.

(**d**) Design of the pyruvate biosensor Pyronic [21]. Binding of pyruvate causes increased fluorescence of cpGFP but not mRuby. Images were created in PowerPoint using built-in elements available within the software.

(**e**) Images of an MCF-10A M3 cell expressing Pyronic and LifeAct-iRFP treated with 50 μM LY294002 at the 0 min (corresponding to **Fig. 4o**, also see **Supplementary Movie 11**). Scale bar: 20 μm.

(**f**) The quantitative responses of 23 individual cells over time in the experiment of **Fig. 4q**.

(**g**) Design of the NADH/NAD+ biosensor Peredox [22]. Binding of NADH causes increased fluorescence of circularly permuted T-Sapphire, a GFP variant with peak excitation around 400 nm. Images were created in PowerPoint using built-in elements available within the software.

(**h**) Images of Peredox/mCherry ratio, mCherry, and Peredox of an MCF-10A M3 cell expressing the NADH/NAD+ biosensor treated with 50 μM LY294002 at 0 min. Scale bar: 20 μm. Also see **Supplementary Movie 12**.

Supplementary Fig. 7

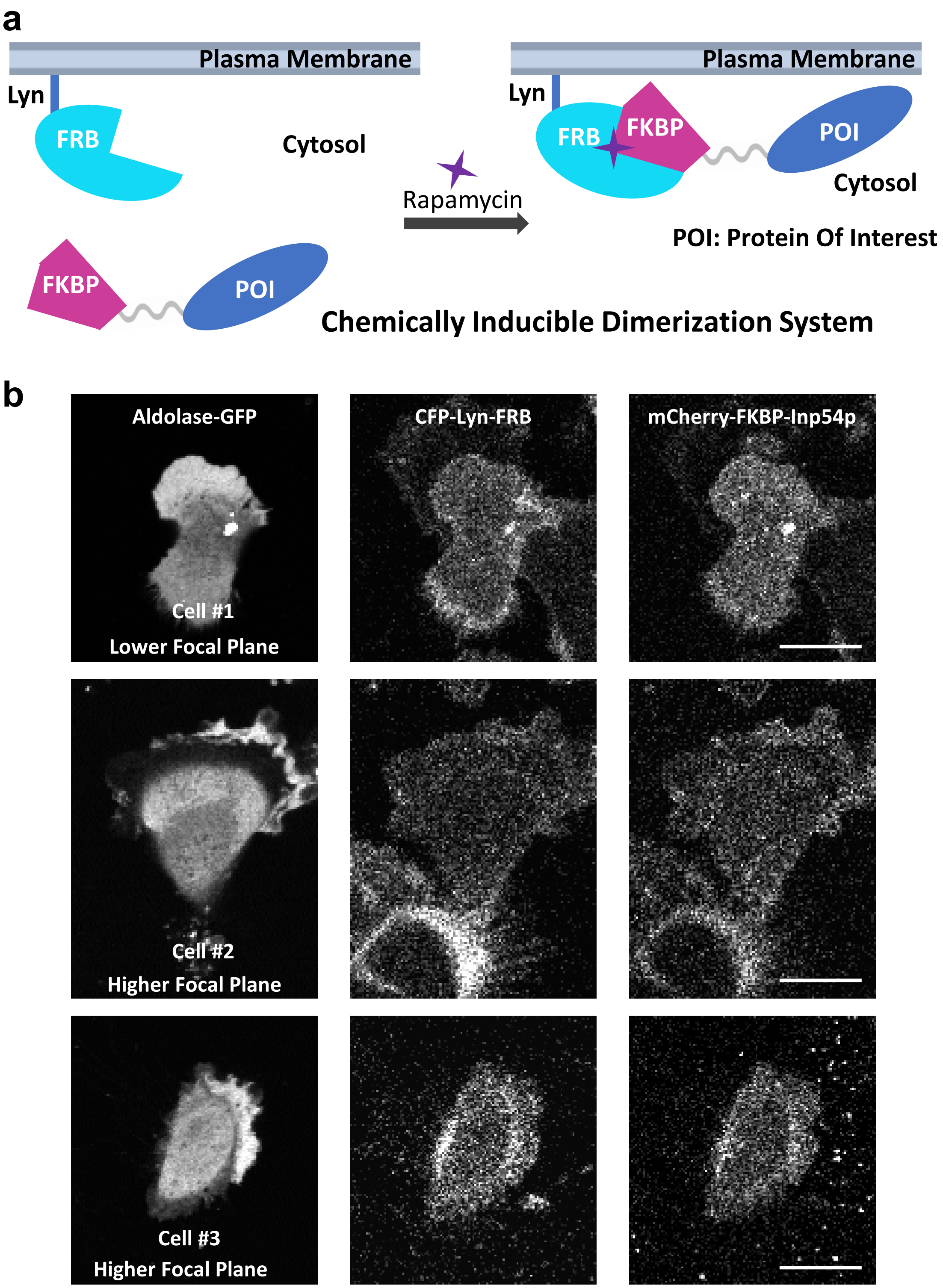

Supplementary Fig. 7 | Enrichment of aldolase in waves and protrusions upon PIP2 depletion.

(**a**) Schematic illustration of chemically induced dimerization (CID) used in **Fig. 5** and **Fig. 6**. Images were created in PowerPoint using built-in elements available within the software.

(**b**) Three more examples of cells with various focal planes showing aldolase-GFP enriched in the spiral peripheral waves and protrusions in MCF10A M1 cells after PIP(4,5)P2 lowering by recruiting the Inp54p to the cell membrane, corresponding to **Fig. 5a, b**. Scale bar: 20 µm.

Supplementary Fig. 8

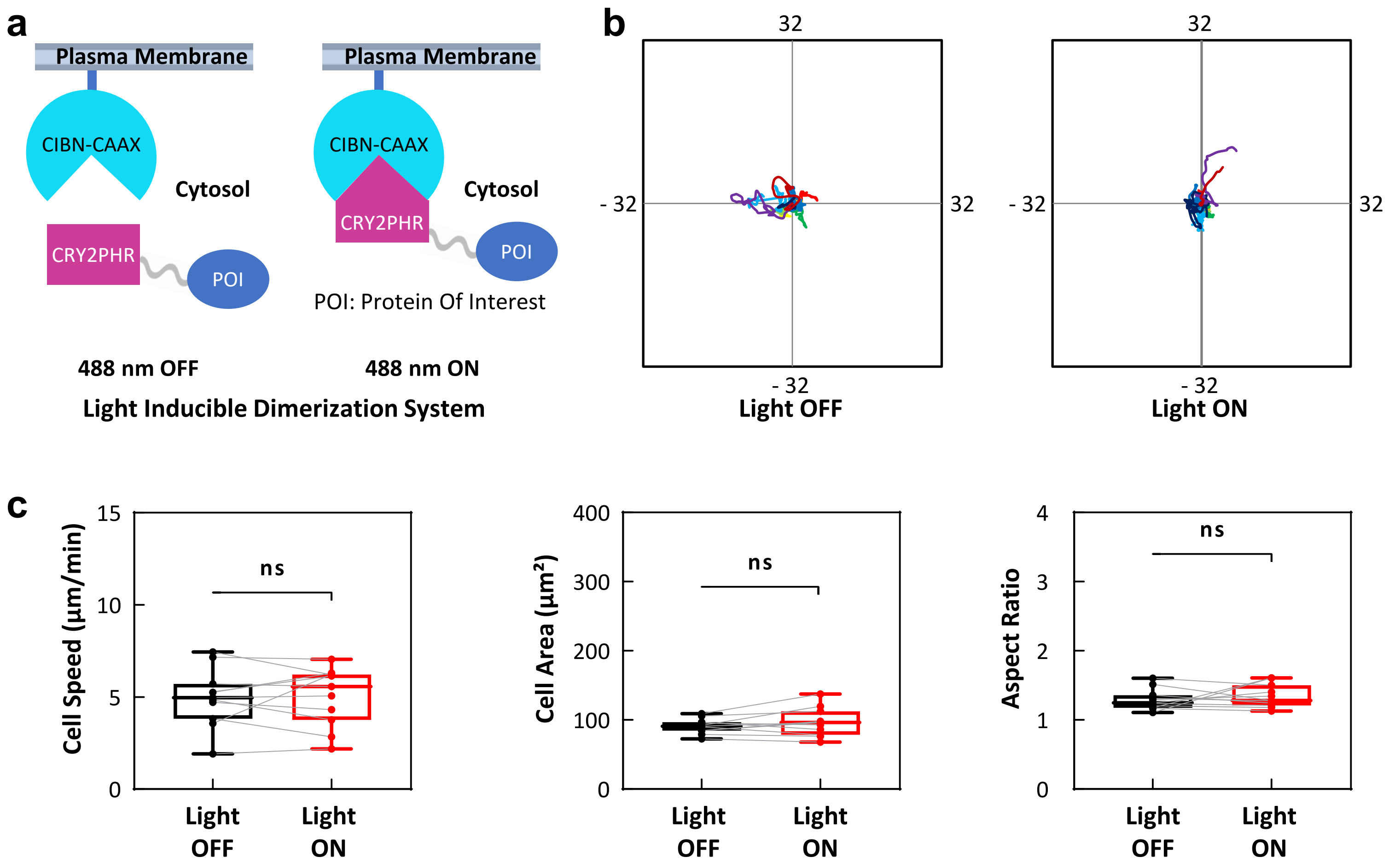

Supplementary Fig. 8 | Optogenetic membrane recruitment of aldolase and controls.

(**a**) Schematic illustration of light inducible dimerization system used in **Fig. 5h-k**. Exposure to 488 nm light triggers a conformational change in CRY2PHR, thereby inducing its binding to CIBN in the plasma membrane. This process brings the POI (aldolase, as shown in **Fig. 5h**) from the cytosol to the cell membrane. Images were created in PowerPoint using built-in elements available within the software.

(**b-c**) Migration tracks (b) and quantification of cell speed, cell area, and aspect ratio (c) in HL-60 cells lacking aldolase recruitment to the plasma membrane, in comparison to the cells shown in **Fig. 5h-k**. n = 11 cells from at least three independent experiments. ns, not significant (Two-tailed paired t test). The boxes extend from 25th to 75th percentiles, median is at the center, and whiskers and outliers are graphed according to Tukey's convention. Connecting lines are provided between paired data points obtained from the same cell, before or after aldolase recruitment.

Supplementary Fig. 9

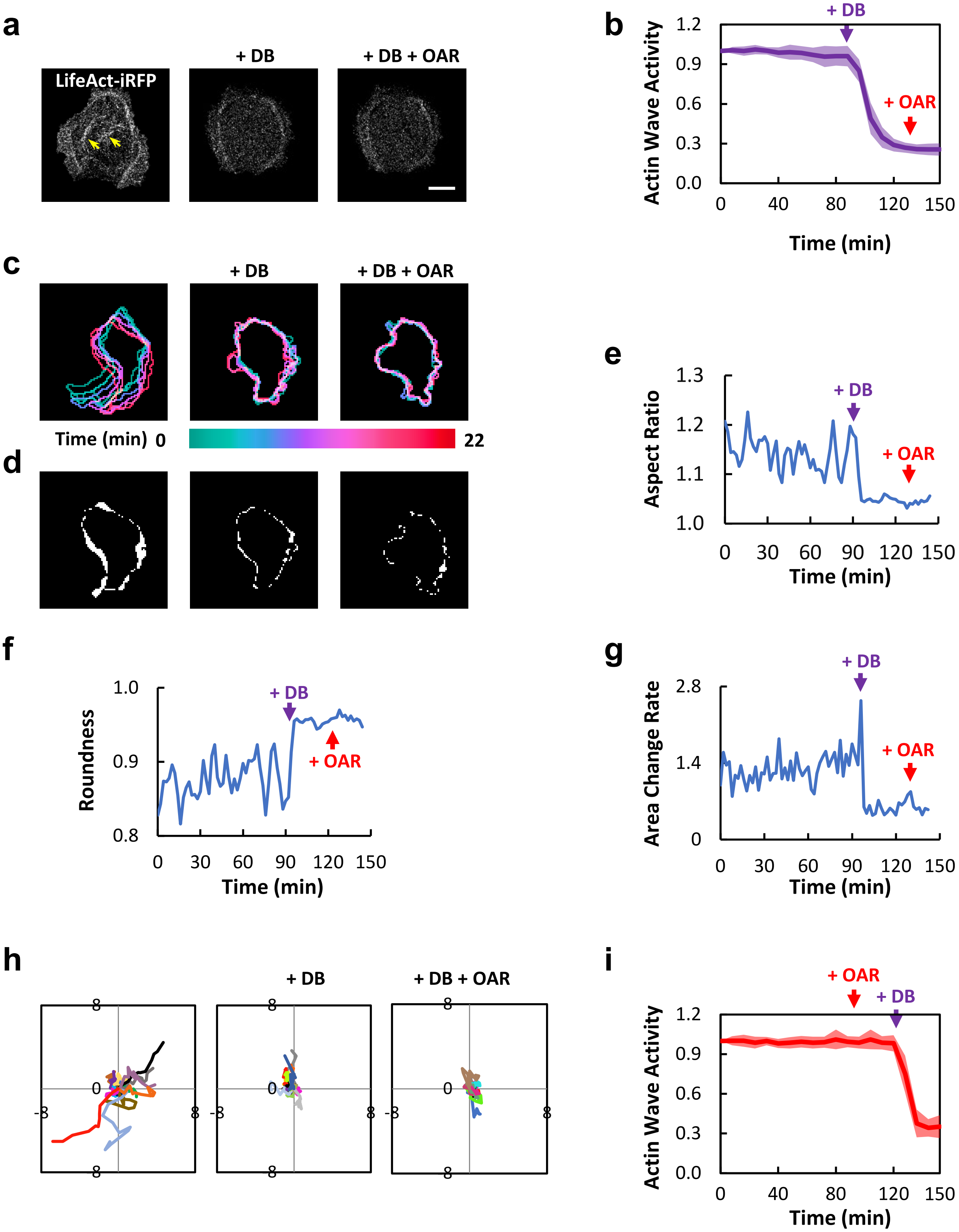

Supplementary Fig. 9 | Effects of glycolysis and OXPHOS inhibition on waves and cell morphodynamics.

(**a**) Images of LifeAct-iRFP in of the cell in **Supplementary Fig. 4c** treated with DB followed by OAR. Yellow arrows indicate the abolish of F-actin waves upon treatment. See also **Supplementary Movie 10**.

(**b**) Actin wave activity, defined as ratio of membrane to cytosol LifeAct intensity, was plotted (mean ± SD) over time for 34 cells from 5 independent experiments treated with DB followed by OAR at the indicated time, corresponding to **Supplementary Fig. 4d**.

(**c**) Color-coded overlays showing the progression of the shape change of an MCF-10A M3 cell over 22 min before and after DB and OAR treatment.

(**d**) Changes in cell morphology between two consecutive frames (3-min timespan) from the time-lapse images in (c).

(**e-g**) Plot of the aspect ratio (e), roundness (f), and the rate of area change (g) over time for the cell in (a).

(**h**) Centroid tracks of 15 cells from 3 independent experiments in (a) showing random motility before and after treatment with DB and OAR.

(**i**) Actin wave activity, defined as ratio of membrane to cytosol LifeAct intensity, was plotted (mean ± SD) over time for 22 cells from 5 independent experiments treated with OAR followed by DB at the indicated time, corresponding to **Supplementary Fig. 4e**.

Supplementary Fig. 10

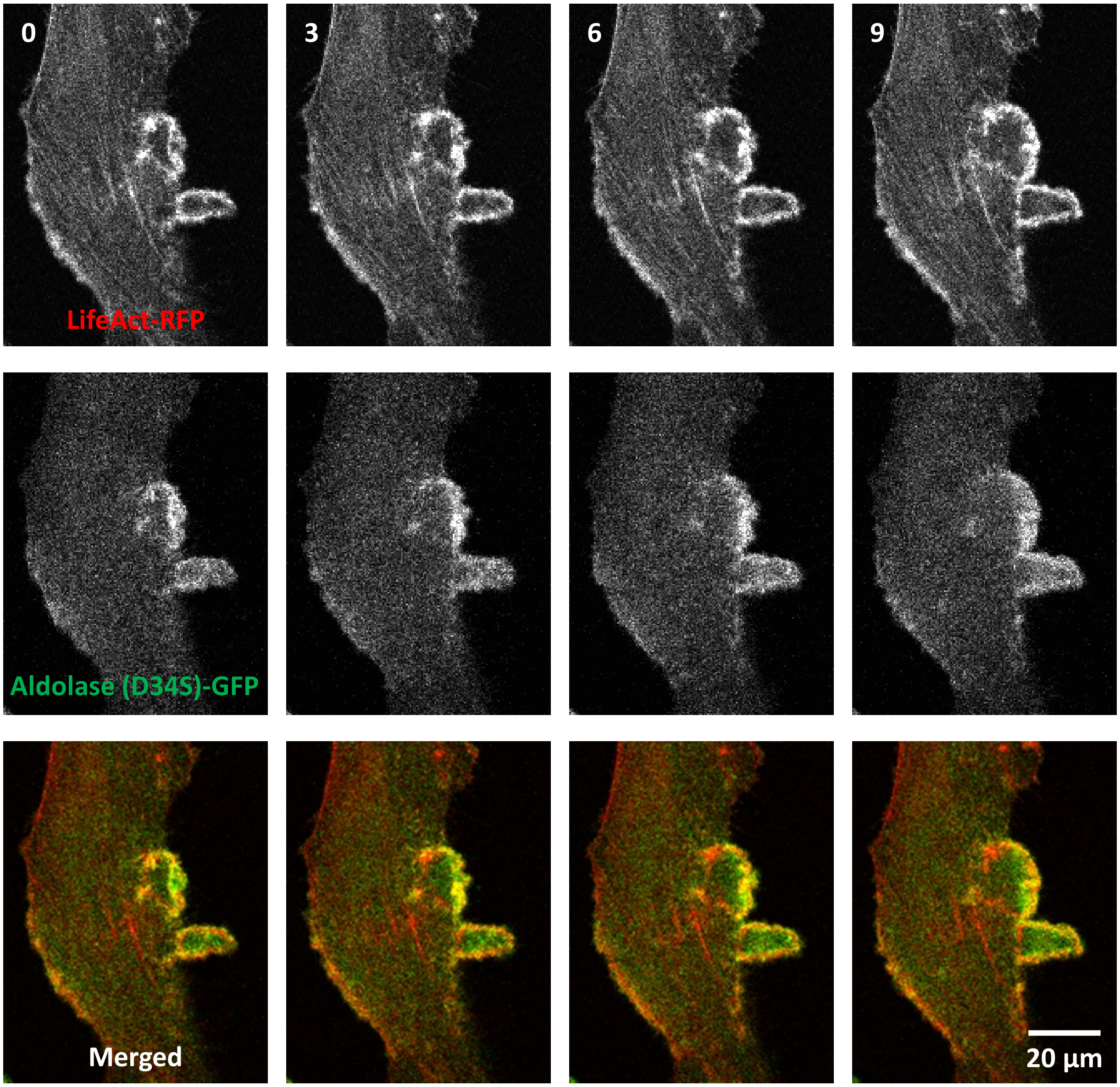

Supplementary Fig. 10 | Catalytic inactive aldolase is still enriched in the waves.

Time-lapse confocal images showing the basal surface of an MCF-10A-M3 cell expressing LifeAct-RFP and Aldolase (D34S)-GFP. Merged images of Aldolase (D34S)-GFP (green) and LifeAct-RFP (red) are also shown. Time stamp is minute and scale bar is 20 µm. These shown images represent a typical example of cells from N ≥ 3 independent experiments.

Supplementary Fig. 11

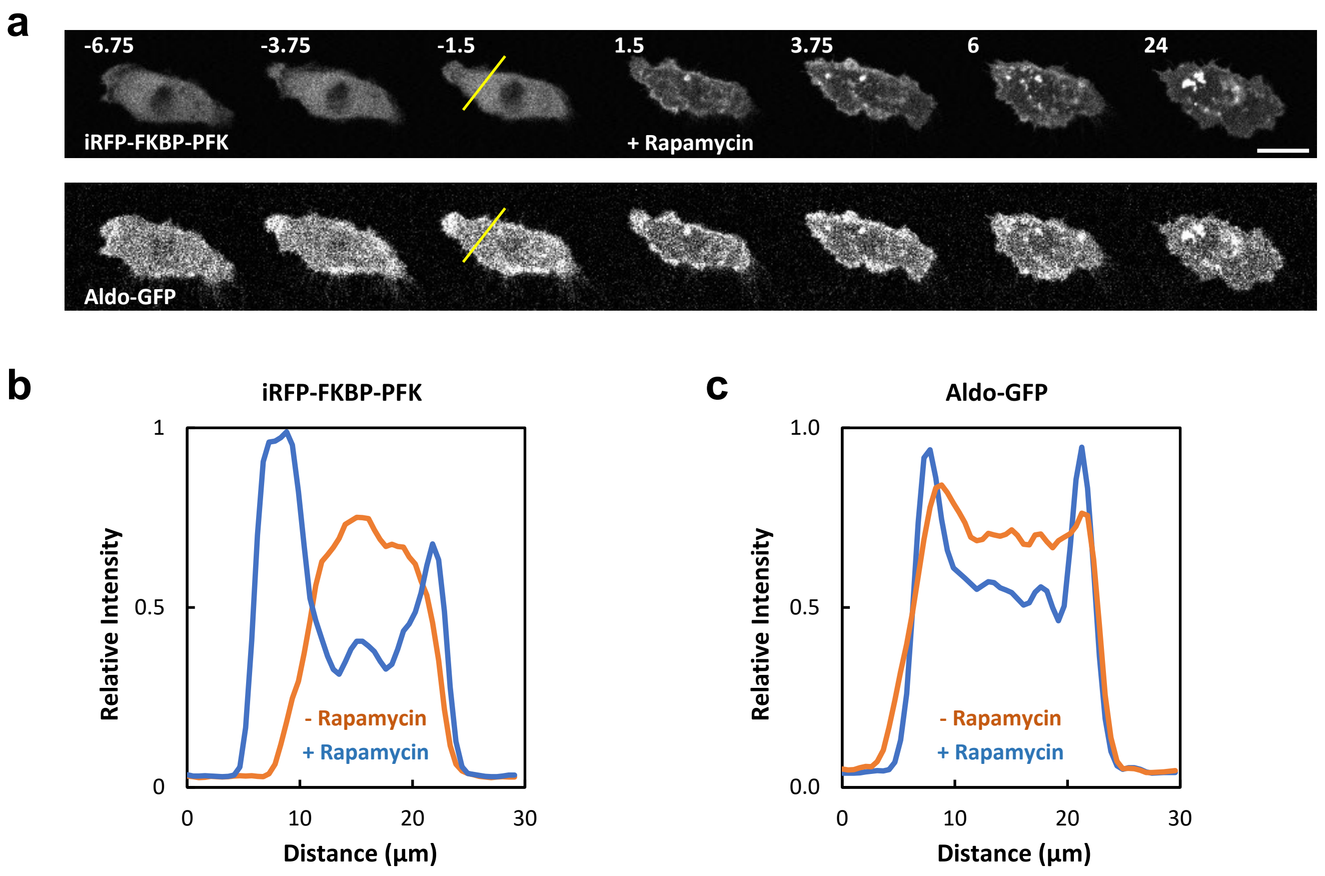

Supplementary Fig. 11 | Effect of PFK membrane recruitment on aldolase localization.

(**a**) Time-lapse confocal images of the iRFP-FKBP-PFK and aldolase-GFP channels of an MCF-10A M3 cell expressing Lyn-FRB, iRFP-FKBP-PFK, and aldolase-GFP treated with 1 μM rapamycin at 0 min, similar to the cell in **Fig. 6b**. Scale bar: 20 μm. Also see **Supplementary Movie 16**.

(**b-c**) Relative intensity of iRFP-FKBP-PFK (b) and aldolase-GFP (c) across the yellow lines in (a) before (orange) and after (blue) rapamycin treatment.

Supplementary Fig. 12

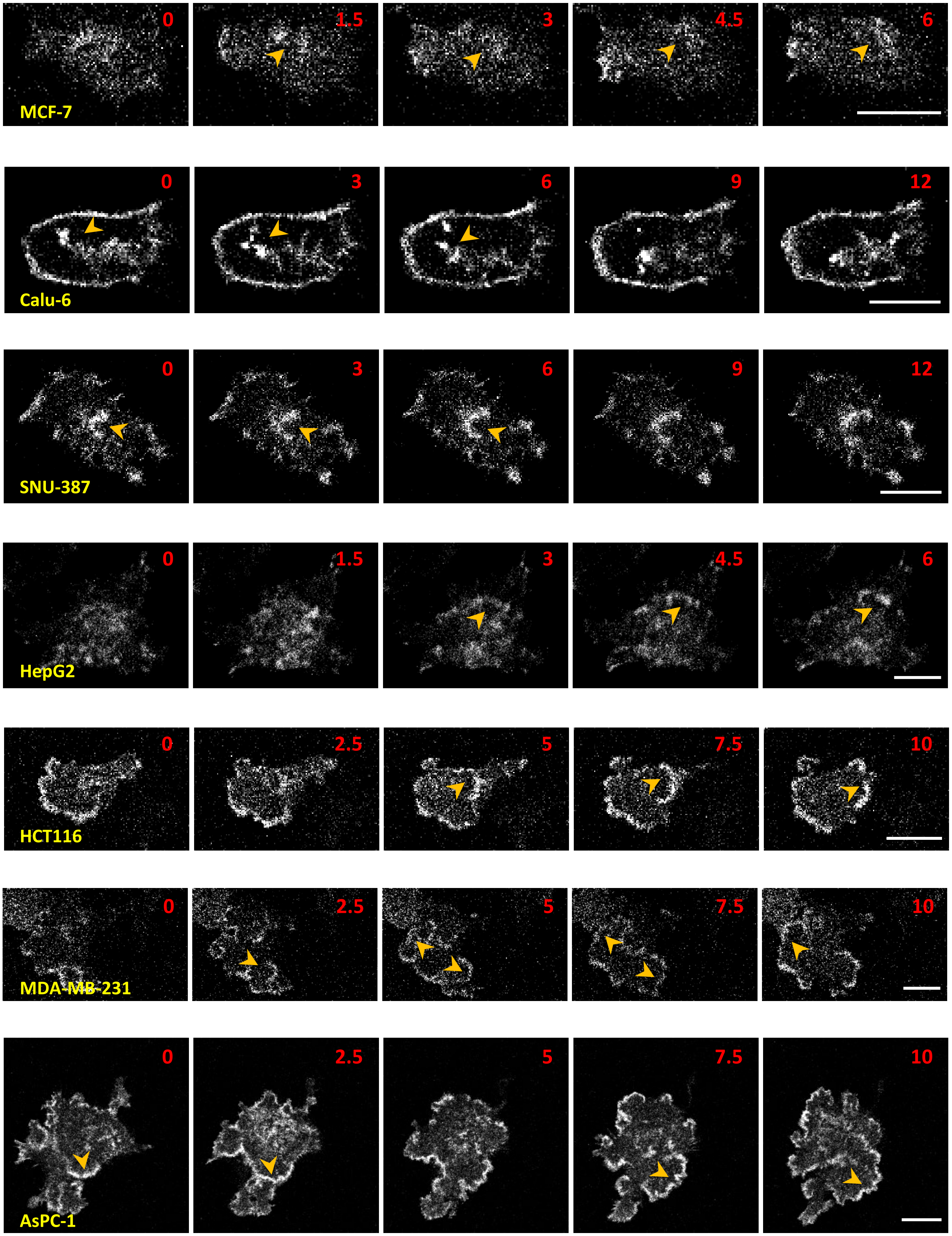

Supplementary Fig. 12 | Different cancer cell lines exhibit varying levels of wave activity.

Time-lapse confocal images of Lifeact-iRFP showing the propagating waves (as indicated by the yellow arrowheads) in 7 cancer cell lines. Time stamp is minute and the scale bar is 20 μm. See also in **Supplementary Movie 17**. The quantification of these wave activities is shown in **Fig. 7d**. These shown images represent a typical example of cells from N ≥ 3 independent experiments.

Supplementary Fig. 13

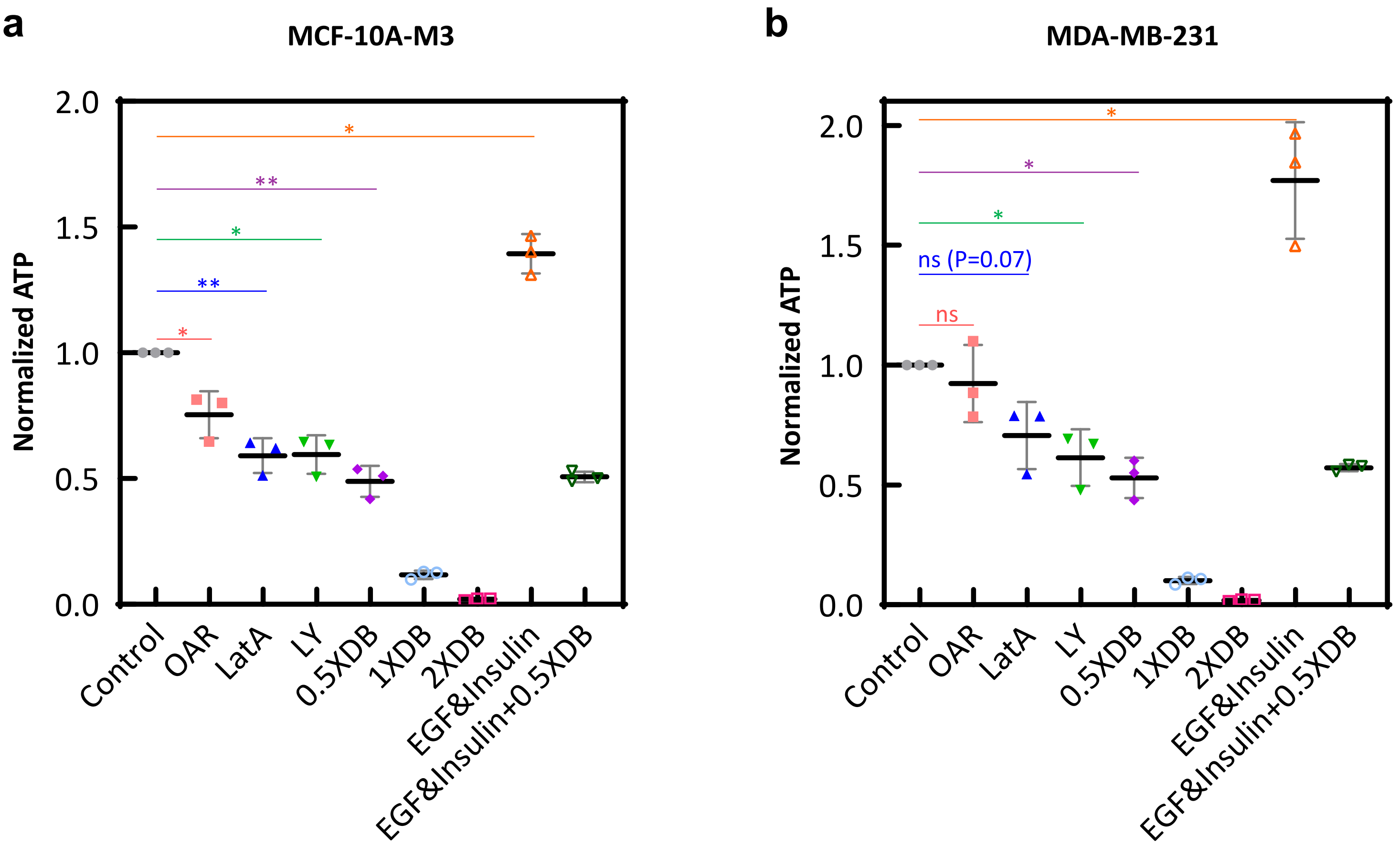

Supplementary Fig. 13 | ATP changes in response to different stimulations or inhibitions were measured using luciferase-based assays.

The mean ± SD of normalized ATP changes in MCF-10A-M3 (a) and MDA-MB-231 (b) cells upon treatment with DMSO, OAR, LatA, LY294002, DB, EGF & insulin, or EGF & insulin plus DB for 40 minutes is shown. The concentrations of inhibitors and growth factors are the same as in previous experiments. Statistical analysis was performed using a two-tailed unpaired t-test with Welch's correction: ****$p < 0.0001$, **$p < 0.01$, $p < 0.05$, ns = not significant. The statistical differences between the DMSO control and OAR, LatA, LY294002, 0.5×DB, and EGF & insulin are indicated by pink, blue, green, purple, and orange statistical symbols ("*" or "ns"), respectively.

Supplementary Fig. 14

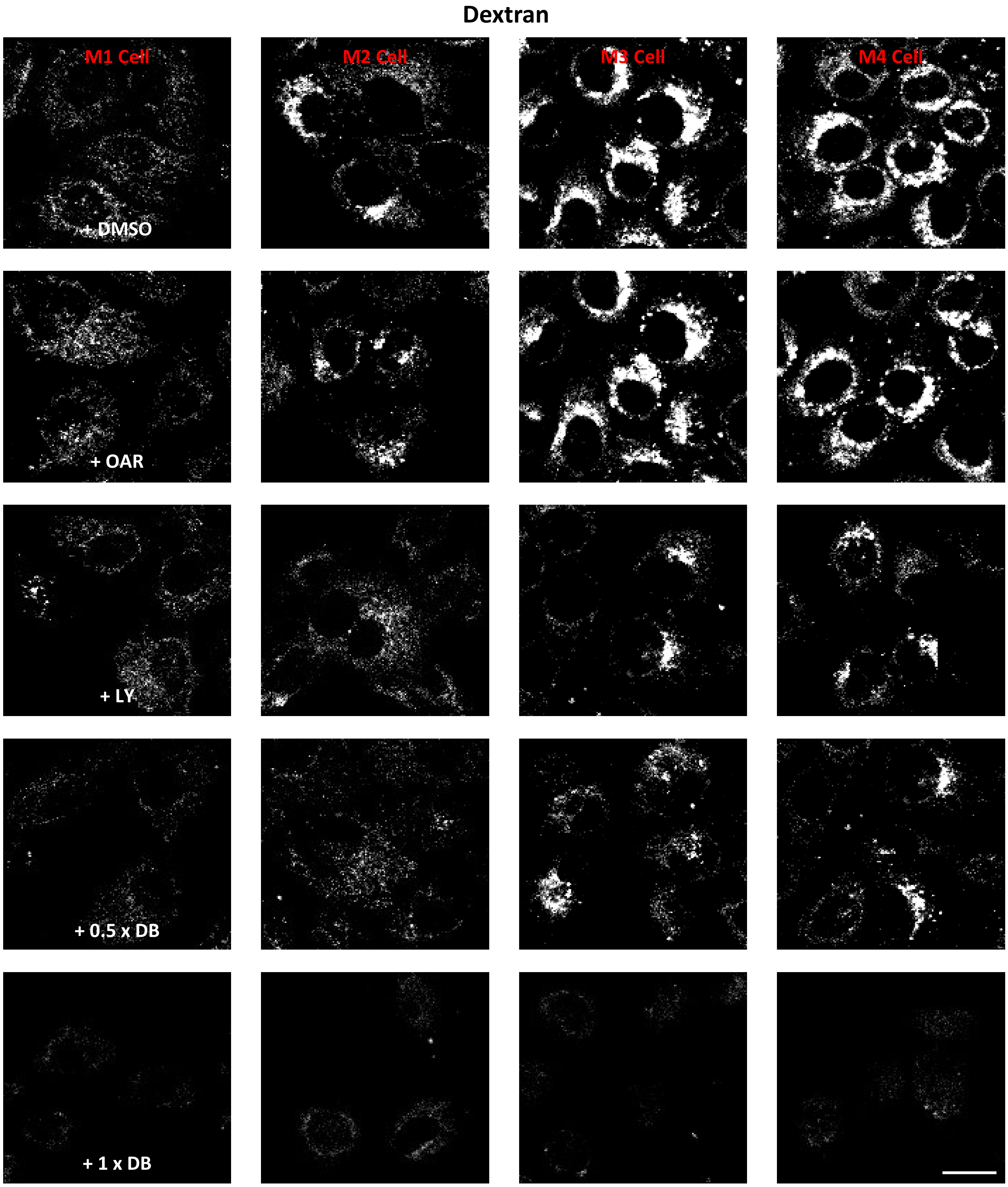

Supplementary Fig. 14 | Uptake of fluorescence conjugated dextran by the M1-M4 cells in different conditions.

Confocal images showing the uptake of fluorescence conjugated dextran by the M1-M4 cells treated with DMSO, OAR, LY, 0.5 x DB, or 1 x DB, respectively. The scale bar is 20 µm. The confocal images with the similar experimental design showing the uptake of fluorescence conjugated BSA by cells is shown in **Fig. 8b**. These shown images represent a typical example of cells from N ≥ 3 independent experiments.

Supplementary Fig. 15

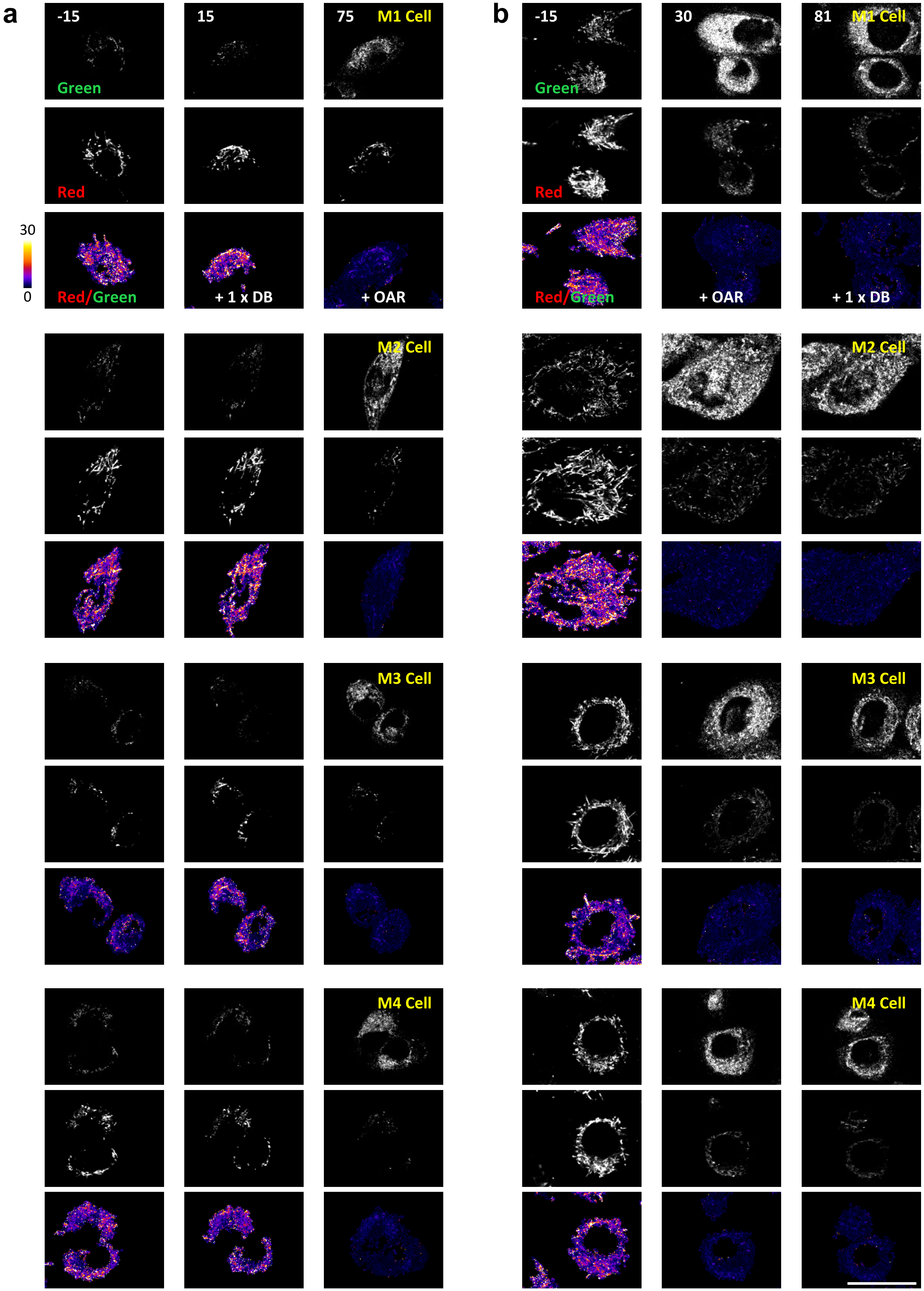

Supplementary Fig. 15 | Mitochondria potential was not affected by the inhibition of glycolytic waves but abolished by the inhibition of OXPHOS.

Time-lapse confocal images of two collected emissions (Green: 515-545 nm, and Red: 570-610 nm) both with 514 nm excitation and ratio images of red/green showing changes of JC-1 dye in the MCF-10A-M3 cells before and after treatment with drugs. 1 x DB was applied at 0 min and OAR was applied at 60 min in (a), while OAR was applied at 0 min and 1 x DB at 60 min in (b). Time stamp is minute and the scale bar is 20 μm. Color scale in all ratio images is 0-30. These shown images represent a typical example of cells from N ≥ 3 independent experiments.
