# Supplementary material for: Self-organizing glycolytic waves tune cellular metabolic states and fuel cancer progression
Source: Nat Commun. 2025 Jul 1;16:5563. doi: 10.1038/s41467-025-60596-6 (PMC12217304; doi:10.1038/s41467-025-60596-6)
Supplement: Supplementary file 2 — Description of Additional Supplementary Files [file 41467_2025_60596_MOESM2_ESM.pdf]

Description of Additional Supplementary Files

File Name: Supplementary Movie 1
Description: *Aldolase is enriched in the propagating waves at the cell membrane/cortex.* Panels from left to right show the aldolase-GFP, NES-mCherry, the merged, and the GFP/mCherry ratio movies of aldolase waves propagating on the basal surface of an MCF-10A M3 cell. Color scale in ratio images is 0-16. Time stamp in the movie is shown as hour:min:sec, which is consistent throughout all the later movies unless otherwise indicated. An individual scale bar is specifically indicated in all movies. Related to Fig. 1a.

File Name: Supplementary Movie 2
Description: *LifeAct is enriched in the propagating waves.* Panels from left to right show the LifeAct-RFP, NES-GFP, the merged, and the RFP/GFP ratio movies of F-actin waves propagating on the basal surface of an MCF-10A M3 cell. Color scale in ratio images is 0-7. Related to Fig. 1c.

File Name: Supplementary Movie 3
Description: *Aldolase is enriched in the LifeAct labelled waves.* Panels from left to right show the aldolase-GFP, LifeAct-RFP, and the merged movies of glycolytic and Factin waves propagating on the basal surface of an MCF-10A M3 cell. Related to Fig. 1e.

File Name: Supplementary Movie 4
Description: *Hexokinase is enriched in the LifeAct labelled waves.* Panels from left to right show the hexokinase-GFP, LifeAct-iRFP, and the merged movies of glycolytic waves propagating on the basal surface of an MCF-10A M3 cell. Related to Fig. 2b.

File Name: Supplementary Movie 5
Description: *Phosphofructokinase (PFK) is enriched in the LifeAct labelled waves.* Panels from left to right show the phosphofructokinase-GFP, LifeAct-iRFP, and the merged movies of glycolytic waves propagating on the basal surface of an MCF-10A M3 cell. Related to Fig. 2d.

File Name: Supplementary Movie 6
Description: *GAPDH is enriched in the LifeAct labelled waves.* Panels from left to right show the GAPDH-RFP, LifeAct-iRFP, and the merged movies of glycolytic waves propagating on the basal surface of an MCF-10A M3 cell. Related to Fig. 2f.

File Name: Supplementary Movie 7
Description: *Enolase is enriched in the LifeAct labelled waves.* Panels from left to right show the enolase-RFP, LifeAct-iRFP, and the merged movies of glycolytic waves propagating on the basal surface of an MCF-10A M3 cell. Related to Fig. 2h.

File Name: Supplementary Movie 8
Description: *Pyruvate kinase is enriched in the LifeAct labelled waves.* Panels from left to right show the pyruvate kinase-RFP, LifeAct-iRFP, and the merged movies of glycolytic waves propagating on the basal surface of an MCF-10A M3 cell. Related to Fig. 2j.

File Name: Supplementary Movie 9
Description: *Waves of aldolase and PFK are increased upon stimulation with growth factors Two.* MCF-10A M3 Cells on top left are expressing aldolase-GFP (related to Fig. 3a) while the two in the bottom left are expressing PFK-GFP (related to Fig. 3d). Glycolytic waves propagating on the basal surface of these MCF-10A M3 cells are imaged before and after treatment with EGF and Insulin. Threshold images indicating the aldolase-GFP wave intensity of aldolase_cell 2 is shown on the right for comparison (related to Fig. 3b, c).

File Name: Supplementary Movie 10
Description: *Effects of glycolysis and OXPHOS inhibitors on intracellular ATP levels and wave activity.* Movie of the basal surface of an MCF-10A M3 cell expressing iATP-cpGFP, mRuby, the cpGFP/mRuby ratio, and LifeAct-iRFP before and after treatment with DB followed by OAR. Color scale in ratio image is 0-1. Related to Supplementary Figs. 4c and 9a.

File Name: Supplementary Movie 11
Description: *Effects of PI3K inhibition on intracellular pyruvate levels and wave activity.* Top row: Movie of the basal surface of an MCF-10A M3 cell expressing pyronic-cpGFP, mRuby, LifeActiRFP, and the cpGFP/mRuby ratio prior to and after the addition of the PI3K inhibitor LY294002. Color scale in ratio image is 0-1 (related to Fig. 4o and Supplementary Fig. 6e). Bottom row: Movie of the basal surface of MCF-10A M3 cells expressing aldolase-GFP and LifeAct-RFP, prior to and after the addition of the PI3K inhibitor LY294002. The merged channel of aldolase (green) and LifeAct (red) are also shown (related to Fig. 4r).

File Name: Supplementary Movie 12
Description: *Effects of wave abolishment by PI3K inhibition on intracellular NADH/NAD+* levels Movie of the basal surface and higher focal plane of an MCF-10A M3 cell expressing peredox/mCherry ratio, peredox, and mCherry prior to and after the addition of the PI3K inhibitor LY294002. Color scale in ratio image is 0-1. Related to Supplementary Fig. 6h.

File Name: Supplementary Movie 13
Description: *Aldolase enriched in the protrusive spiral waves upon acute PI(4,5)P2 reduction on the plasma membrane.* Movie showing the recruitment of aldolase-GFP (channel shown in the movie) to the protrusive spiral waves in a previously quiescent MCF-10A cell after the addition of rapamycin, which induces the recruitment of FKBP-Inp54p to the plasma membrane and thus lowering of PI(4,5)P2. Related to Fig. 5b.

File Name: Supplementary Movie 14
Description: *Effect of synthetic recruitment of PFK from cytosol to the plasma membrane.* Movie showing the recruitment of GFP-FKBP-PFK (left) after the addition of rapamycin initiates cell spreading and enhances dynamic F-actin patch activities labeled by LifeAct-iRFP (right) in an MCF-10A M3 cell. Related to Fig. 5e.

File Name: Supplementary Movie 15
Description: *Effect of optogenetic recruitment of aldolase to the plasma membrane in neutrophil cells.* Movie showing a differentiated HL-60 neutrophil expressing CIBN-CAAX, CRY2PHR-mCherry-aldolase and LifeAct-miRFP703, before and after 488 nm light

illumination. The HL-60 cell becomes highly motile and polarized after aldolase recruitment to the plasma membrane. Related to Fig. 5h.

File Name: Supplementary Movie 16
Description: *Effect of membrane recruitment of PFK on the localization of aldolase*. Movie of two different MCF-10A M3 cells with 3 different focal planes showing the translocation of iRFPFKBP-PFK (row 1 and 3) after addition of rapamycin induces cell spreading and the recruitment of aldolase-GFP (row 2 and 4) to the plasma membrane. Related to Fig. 6b and Supplementary Fig. 11a.

File Name: Supplementary Movie 17
Description: *Different levels of wave activity in a series of cancer cell lines derived from various tissues.* Movie of LifeAct at the cellular basal surface showing the propagating waves in MCF-7, Calu-6, SNU-387, HepG2, HCT116, MDA-MB-231, and AsPC-1 cells, respectively. Related to Fig. 7d and Supplementary Fig. 12.
