# Supplementary material for: Self-organizing glycolytic waves tune cellular metabolic states and fuel cancer progression
Source: Nat Commun. 2025 Jul 1;16:5563. doi: 10.1038/s41467-025-60596-6 (PMC12217304; doi:10.1038/s41467-025-60596-6)
Supplement: Supplementary file 20 — Reporting Summary [file 41467_2025_60596_MOESM20_ESM.pdf]

nature portfolio

Corresponding author(s): Huiwang Zhan, Chris Janetopoulos, Chuan-Hsiang Huang, Peter N. Devreotes

Last updated by author(s): May 20, 2025

Reporting Summary

Nature Portfolio wishes to improve the reproducibility of the work that we publish. This form provides structure for consistency and transparency in reporting. For further information on Nature Portfolio policies, see our Editorial Policies and the Editorial Policy Checklist.

Statistics

For all statistical analyses, confirm that the following items are present in the figure legend, table legend, main text, or Methods section.

n/a	Confirmed	
☐	☒	The exact sample size (n) for each experimental group/condition, given as a discrete number and unit of measurement
☐	☒	A statement on whether measurements were taken from distinct samples or whether the same sample was measured repeatedly
☐	☒	The statistical test(s) used AND whether they are one- or two-sided<br>*Only common tests should be described solely by name; describe more complex techniques in the Methods section.*
☒	☐	A description of all covariates tested
☐	☒	A description of any assumptions or corrections, such as tests of normality and adjustment for multiple comparisons
☐	☒	A full description of the statistical parameters including central tendency (e.g. means) or other basic estimates (e.g. regression coefficient) AND variation (e.g. standard deviation) or associated estimates of uncertainty (e.g. confidence intervals)
☐	☒	For null hypothesis testing, the test statistic (e.g. F, t, r) with confidence intervals, effect sizes, degrees of freedom and P value noted<br>*Give P values as exact values whenever suitable.*
☒	☐	For Bayesian analysis, information on the choice of priors and Markov chain Monte Carlo settings
☒	☐	For hierarchical and complex designs, identification of the appropriate level for tests and full reporting of outcomes
☒	☐	Estimates of effect sizes (e.g. Cohen's d, Pearson's r), indicating how they were calculated

Our web collection on statistics for biologists contains articles on many of the points above.

Software and code

Policy information about availability of computer code

Data collection	Confocal image videos were acquired using Zeiss LSM 800 and LSM 780 microscopes, operated with ZEN Blue (v2.3) or ZEN Black (v2.3 SP1 FP2) software. Wide-field fluorescence imaging was performed on a Nikon Ti2-E microscope equipped with an iLas2 Ring-TIRF module and controlled via NIS-Elements software (AR 4.40.00, Build 1084). Biochemical measurement of ATP was conducted using a luminescent detection assay kit and recorded with a FLUOstar Omega microplate reader (BMG Labtech).
Data analysis	Fiji/ImageJ 1.52i, GraphPad Prism 9.2.0, and Microsoft Excel 2019 softwares were used for all data analysis. Any additional information will be available from the corresponding authors upon request.

For manuscripts utilizing custom algorithms or software that are central to the research but not yet described in published literature, software must be made available to editors and reviewers. We strongly encourage code deposition in a community repository (e.g. GitHub). See the Nature Portfolio guidelines for submitting code & software for further information.

Data

Policy information about availability of data

All manuscripts must include a data availability statement. This statement should provide the following information, where applicable:

- Accession codes, unique identifiers, or web links for publicly available datasets
- A description of any restrictions on data availability
- For clinical datasets or third party data, please ensure that the statement adheres to our policy

All raw data and associated statistical analyses are provided with this study. The wave data shown in Fig. 7a were reanalyzed and replotted from our previous publication (PMID: 32877650). All other data supporting the findings of this study are available from the corresponding authors upon request. Source data are provided with this paper.

Research involving human participants, their data, or biological material

Policy information about studies with human participants or human data. See also policy information about sex, gender (identity/presentation), and sexual orientation and race, ethnicity and racism.

Reporting on sex and gender	Not Applicable.
Reporting on race, ethnicity, or other socially relevant groupings	Not Applicable.
Population characteristics	Not Applicable.
Recruitment	Not Applicable.
Ethics oversight	Not Applicable.

Note that full information on the approval of the study protocol must also be provided in the manuscript.

Field-specific reporting

Please select the one below that is the best fit for your research. If you are not sure, read the appropriate sections before making your selection.

☒ Life sciences ☐ Behavioural & social sciences ☐ Ecological, evolutionary & environmental sciences

For a reference copy of the document with all sections, see nature.com/documents/nr-reporting-summary-flat.pdf

Life sciences study design

All studies must disclose on these points even when the disclosure is negative.

Sample size	Sample sizes were selected to appropriately account for cellular heterogeneity. Although no statistical methods were used to pre-determine these sizes, they are comparable to those reported in previous studies from our laboratory (Lampert T et al., 2017; Miao Y et al., 2019; Zhan H et al., 2020; Pal D et al., 2023; Banerjee T et al., 2023). Additionally, the selected sample sizes are consistent with those reported by other researchers in the field (Yang Y et al., 2017; Neumann NM et al., 2018; Tsujita K et al., 2021; Bell GR et al., 2021; Riedl M et al., 2023; Hu M et al., 2024). Similar sample sizes were applied to both experimental and control groups. All experiments were independently repeated at least three times, often using parallel imaging setups. For each plot, a minimum of 200 images was typically available for analysis.
Data exclusions	No data were excluded from the analyses.
Replication	All reported findings were reliably reproduced using at least three independent biological replicates.
Randomization	This study did not involve any human or animal subjects. For all experimental and control groups used in imaging assays, cells were randomly selected from parental cell lines in culture. For experiments such as drug treatments, cells were randomized into wells.
Blinding	Blinding during data collection was sometimes not feasible, as differences in protein expression levels, localization, or dynamics between experimental and control groups were often visually apparent under the microscope. However, readouts were not subject to experimenter bias. Blinding was not relevant during data analysis, as the majority of quantifications were performed using automated image analysis in Fiji/ImageJ, with consistent quantification parameters applied across all experimental and control groups.

Reporting for specific materials, systems and methods

We require information from authors about some types of materials, experimental systems and methods used in many studies. Here, indicate whether each material, system or method listed is relevant to your study. If you are not sure if a list item applies to your research, read the appropriate section before selecting a response.

Materials & experimental systems

n/a	Involved in the study	
☐	☒	Antibodies
☐	☒	Eukaryotic cell lines
☒	☐	Palaeontology and archaeology
☒	☐	Animals and other organisms
☒	☐	Clinical data
☒	☐	Dual use research of concern
☒	☐	Plants

Methods

n/a	Involved in the study	
☒	☐	ChIP-seq
☒	☐	Flow cytometry
☒	☐	MRI-based neuroimaging

Antibodies

Antibodies used	Primary antibodies against GFP (Invitrogen, #33-2600), GAPDH (Invitrogen, #MA5-15738), Aldolase A (Sigma-Aldrich, #HPA004177), Enolase 1 (Proteintech, #11204-1-AP), PFKP (Sigma-Aldrich, # HPA018257), and HK-1 (Proteintech, #19662-1-AP). Secondary antibodies: Goat anti-Mouse IgG (H+L) Alexa Fluor™ 660 (Invitrogen, #A-21055), Goat anti-Rabbit IgG (H+L) Alexa Fluor™ 633 (Invitrogen, # A-21071), Goat anti-Mouse IgG (H+L) Alexa Fluor™ 488 (Invitrogen, #A-11001), and Goat anti-Rabbit IgG (H+L) Alexa Fluor™ 488 (Invitrogen, #A-11034).
Validation	The anti-GFP antibody (Invitrogen, #33-2600) is validated for Western blot (WB), immunofluorescence (IF), and immunohistochemistry (IHC), and widely cited for detecting GFP-tagged proteins.<br><br>The anti-GAPDH antibody (Invitrogen, #MA5-15738) is validated for WB, ICC, IF, IHC, flow cytometry, and ELISA across multiple species.<br><br>The anti-Aldolase A antibody (Sigma-Aldrich, #HPA004177) is validated for WB, IF, and IHC, with supporting data from the Human Protein Atlas.<br><br>The anti-Enolase 1 antibody (Proteintech, #11204-1-AP) is validated for WB, IF/ICC, IHC, flow cytometry, IP, and ELISA, with published images.<br><br>The anti-PFKP antibody (Sigma-Aldrich, #HPA018257) is validated for WB, IF, and IHC in human samples, as part of the Prestige Antibody® initiative.<br><br>The anti-HK1 antibody (Proteintech, #19662-1-AP) is validated for WB, IF/ICC, and IP, with vendor-provided immunofluorescence and WB data.<br><br>The Alexa Fluor 660 goat anti-mouse secondary antibody (Invitrogen, #A-21055) is highly cross-adsorbed and validated for IF, flow cytometry, and ICC.<br><br>The Alexa Fluor 633 goat anti-rabbit secondary antibody (Invitrogen, #A-21071) is cross-adsorbed and validated for IF, WB, and flow cytometry.<br><br>The Alexa Fluor 488 goat anti-mouse secondary antibody (Invitrogen, #A-11001) is validated for IF and flow cytometry, with reduced cross-reactivity.<br><br>The Alexa Fluor 488 goat anti-rabbit secondary antibody (Invitrogen, #A-11034) is validated for IF and IHC, with high specificity and cross-adsorption.

Eukaryotic cell lines

Policy information about cell lines and Sex and Gender in Research

Cell line source(s)	M1 (MCF-10A), M2 (MCF-10AT1k.cl2), M3 (MCF-10CA1h), and M4 (MCF-10CA1a.cl1) cells were obtained from the Animal Model and Therapeutic Evaluation Core (AMTEC) at the Karmanos Cancer Institute, Wayne State University. The MCF-7 cell line was obtained from the Huang Lab (JHU); AsPC-1 from the Doug Robinson Lab (JHU); and SNU-387, Calu-6, HepG2, MDA-MB-231, and HCT116 from the Jun Liu Lab (JHU). The human HL-60 cell line (ATCC #CCL-240; RRID:CVCL_0002) was provided by the Orion Weiner Lab (UCSF).
Authentication	M1 (MCF-10A), M2 (MCF-10AT1k.cl2), M3 (MCF-10CA1h), and M4 (MCF-10CA1a.cl1) cells were authenticated by the Animal Model and Therapeutic Evaluation Core (AMTEC) at the Karmanos Cancer Institute, Wayne State University. HL-60, MCF-7, AsPC-1, SNU-387, Calu-6, HepG2, MDA-MB-231, and HCT116 cell lines were originally authenticated by their respective suppliers using morphology and short tandem repeat (STR) profiling. All cell lines maintained in culture exhibited the expected morphology and growth characteristics.
Mycoplasma contamination	No mycoplasma contamination was detected.

Commonly misidentified lines<br>(See ICLAC register)	No cell lines used in this study are listed in ICLAC register. No commonly misidentified cell lines were used in this study.

Plants

Seed stocks	*Report on the source of all seed stocks or other plant material used. If applicable, state the seed stock centre and catalogue number. If plant specimens were collected from the field, describe the collection location, date and sampling procedures.*
Novel plant genotypes	*Describe the methods by which all novel plant genotypes were produced. This includes those generated by transgenic approaches, gene editing, chemical/radiation-based mutagenesis and hybridization. For transgenic lines, describe the transformation method, the number of independent lines analyzed and the generation upon which experiments were performed. For gene-edited lines, describe the editor used, the endogenous sequence targeted for editing, the targeting guide RNA sequence (if applicable) and how the editor was applied.*
Authentication	*Describe any authentication procedures for each seed stock used or novel genotype generated. Describe any experiments used to assess the effect of a mutation and, where applicable, how potential secondary effects (e.g. second site T-DNA insertions, mosiacism, off-target gene editing) were examined.*
